# Supplementary material for: Clinical Features of Human Parvovirus B19-Associated Encephalitis Identified in the Dakar Region, Senegal, and Viral Genome Characterization
Source: Viruses. 2025 Jan 15;17(1):111. doi: 10.3390/v17010111 (PMC11769063; doi:10.3390/v17010111)
Supplement: Supplementary file 1 [file viruses-17-00111-s001.zip › viruses-3313089-supplementary.pdf]

Table S1. PCR and Sequencing of parvovirus B19 from CSF samples

Sample identification	Date of collection	Type of sample	RT-PCR results	Ct value	Genbank accession numbers
**395961**	24/06/2022	CSF; NP; Serum	B19V	32.05	
**395992**	16/07/2022	CSF; Serum	B19V	39.34	
**408220**	27/09/2022	CSF	B19V	40.12	
**408241**	06/10/2022	CSF; NP	B19V	38.31	
**408267**	17/10/2022	CSF	B19V; HSV-1	34.8	
**408344**	24/11/2022	CSF	B19V	38.08	
**415297**	10/03/2023	CSF	B19V	31.36	
**417403**	18/04/2023	CSF	B19V	29.8	
**417406**	18/04/2023	CSF	B19V	33.36	PQ390712
**417407**	19/04/2023	CSF	B19V	36.07	
**417411**	19/04/2023	CSF	B19V	31.7	
**417430**	28/04/2023	CSF	B19V	31.09	PQ390713
**417438**	02/05/2023	CSF	B19V	28.8	PQ390714

Ct: threshold cycle; NP: nasopharyngeal; CSF: cerebrospinal fluid
